# Docosahexaenoic acid inhibits zymogen activation by suppressing vacuolar ATPase activation in cerulein-stimulated pancreatic acinar cells

**DOI:** 10.1186/s12263-020-00664-2

**Published:** 2020-03-23

**Authors:** Yeeun Park, Leeyeon Ku, Joo Weon Lim, Hyeyoung Kim

**Affiliations:** Department of Food and Nutrition, Brain Korea 21 PLUS Project, College of Human Ecology, Yonsei University, Seoul, 03722 Korea

**Keywords:** Cerulein, Docosahexaenoic acid (DHA), Pancreatic acinar cells, Parkin, Vacuolar ATPase (vATPase), Zymogen

## Abstract

**Background:**

The premature activation of digestive enzyme zymogens within pancreatic acinar cells is an important early feature of acute pancreatitis. Supraphysiological concentrations of cholecystokinin (CCK) cause intrapancreatic zymogen activation and acute pancreatitis. Stimulation of vacuolar ATPase (vATPase) activity is required for zymogen activation in pancreatic acinar cells. Parkin, a multiprotein E3 ubiquitin ligase complex, promotes vATPase ubiquitination and degradation, which inhibits vATPase activity. Docosahexaenoic acid (DHA), an omega-3 fatty acid, exerts anti-inflammatory effects. It is reported to bind to G-protein coupled receptor 120 (GPR120) and GPR40. DHA induces the degradation of certain proteins by activating ubiquitin-proteasome system in various cells. This study aimed to investigate whether DHA induces Parkin and inhibits vATPase activity, resulting in zymogen inactivation in pancreatic acinar AR42J cells stimulated with cerulein, a CCK analog.

**Results:**

Cerulein induced the translocation of the cytosolic V1 domain (E subunit) of vATPase to the membrane, which indicated vATPase activation, and zymogen activation in AR42J cells. DHA suppressed the association of the vATPase with membranes, and zymogen activation (increased trypsin activity and amylase release) induced by cerulein. Pretreatment with a GPR120 antagonist AH-7614, a GPR40 antagonist DC260126, or an ubiquitination inhibitor PYR-41 reduced the effect of DHA on cerulein-induced zymogen activation. Treatment with PYR-41 reversed the DHA-induced decrease in vATPase activation in cerulein-treated cells. Furthermore, DHA increased the level of Parkin in membranes of cerulein-treated cells.

**Conclusions:**

DHA upregulates Parkin which inhibits vATPase-mediated zymogen activation, via GPR120 and GPR40, in cerulein-stimulated pancreatic acinar cells.

## Background

The pancreatic acinar cell, which comprises over 90% of the exocrine pancreas, synthesizes and secretes the enzymes required to digest nutrients. Many of these digestive enzymes are stored in acinar cells as inactive zymogens that become activated only after reaching the small intestine. Premature activation of these zymogens within acinar cells appears to have a critical role in initiating acute pancreatitis [1], which can result in autodigestion of the pancreas and multiple organ dysfunction. It is also associated with increased cytokine production and release, ultimately leading to deleterious local and systemic effects [2–4]. Similar symptoms are observed in cerulein pancreatitis, one of the best characterized and widely used experimental animal models of pancreatitis. Supramaximal doses of cerulein, a cholecystokinin (CCK) analog, result in experimental pancreatitis [5, 6]. Doses of cerulein beyond those that cause the maximum pancreatic secretion of amylase and lipase [7, 8] result in pancreatitis, which is characterized by a dysregulation of the production and secretion of digestive enzymes. In particular, pancreatitis is characterized by abnormal pancreatic secretion and an elevation in the serum levels of digestive enzymes, cytoplasmic vacuolization, and the death of acinar cells, edema formation, and an infiltration of inflammatory cells into the pancreas [9, 10]. The mortality rate in patients with acute pancreatitis has decreased over the last decade due to improvements in critical care. However, the worldwide incidence of acute pancreatitis remains high [11].

Although the mechanisms involved in the pathogenesis of pancreatitis are not completely elucidated, the premature activation of zymogens in pancreatic acinar cells is regarded as a major pathogenic factor in acute pancreatitis [12, 13]. Induction of experimental pancreatitis in rat pancreatic acini with supraphysiological doses of cerulein causes an increase in intracellular zymogen activation [14].

Vacuolar ATPase (vATPase) is an ATP-dependent proton pump found within the membranes of many organelles [15]. vATPase is composed of an integral membrane domain (V0) and a peripheral complex (V1). The V1 domain must be transported to the membrane and assemble with the V0 domain to activate vATPase [16]. Cerulein is known to induce the activation of vATPase in pancreatic acini [17]. The V1 domain of vATPase consists of eight subunits, A–H. Translocation of the E subunit to the membrane activates vATPase in pancreatic acinar cells [17] and pancreatic cancer cells [18]. Therefore, translocation of the E subunit from the cytosol to the membrane was used an index of vATPase activation in cerulein-treated pancreatic acinar cells in the present study.

It was recently reported that vATPase activity is required for zymogen activation in pancreatic acinar cells [19]. Considerable evidence indicates that cerulein stimulates zymogen activation and that specific inhibitors of vATPase, such as bafilomycin A1 and concanamycin A, inhibit cerulein-induced zymogen activation. Martinez et al. [20] reported that vATPase could be ubiquitinated by Parkin. These data suggest that Parkin may protect the cells against premature zymogen activation by reducing vATPase activation through the induction of proteasome-mediated degradation.

Docosahexaenoic acid (DHA), an omega-3 polyunsaturated fatty acid (PUFA), is the longest and the most unsaturated fatty acid, with 22 carbons and 6 double bonds (C22:6n-3). DHA has been known to exert anti-inflammatory and anti-oxidant effects, resulting in the prevention of various diseases, including cardiovascular diseases and autoimmune diseases [21]. DHA is known to act as endogenous ligand of G-protein coupled receptor 120 (GPR120) [22] and GPR40 (free fatty acid receptor 1) [23]. DHA exerts anti-inflammatory effects via the GPR-120-mediated pathway in various cell lines [24]. In animal experiment, DHA inhibited a complete Freund’s adjuvant (CFA)-induced inflammatory chronic pain via GPR40 signaling pathway [25]. Moreover, recent studies indicate that DHA can induce the degradation of some proteins by activating the ubiquitin-proteasome system in cells [26, 27]. Therefore, we hypothesize that DHA may prevent acute pancreatitis by inducing Parkin-mediated degradation of vATPase and thus, reducing zymogen activation in pancreatic acinar cells.

The aim of this study was to investigate whether DHA inhibits zymogen activation by upregulating Parkin and inhibiting vATPase activation in cerulein-stimulated pancreatic acinar AR42J cells.

## Results

### Cerulein activated the E subunit of vATPase in AR42J cells

**Fig. 1:**
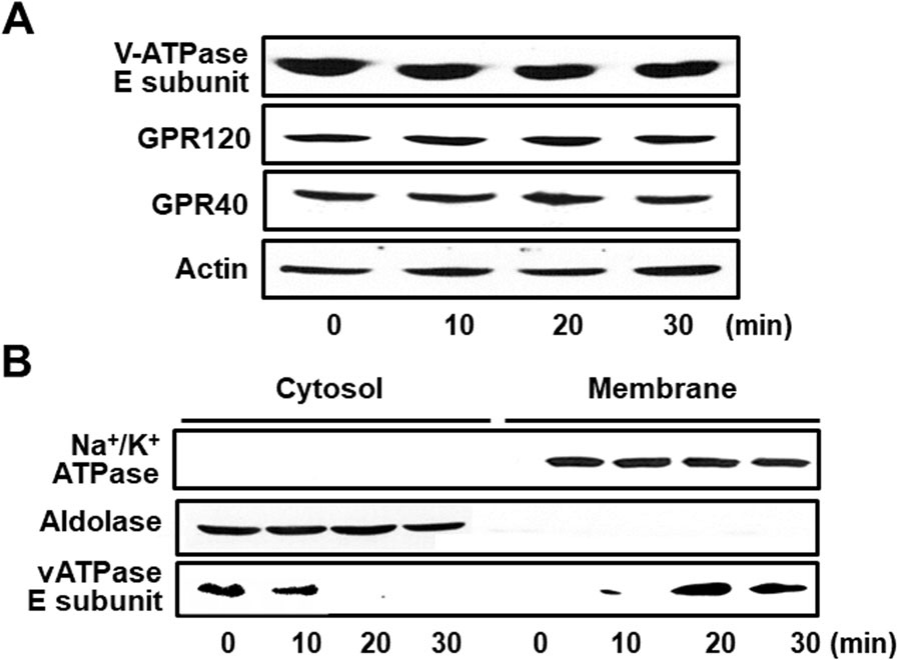
Cerulein-induced translocation of the E subunit of vATPase to the membrane. Cells were stimulated with cerulein (10^−8^ M) for the indicated time periods. **a** Levels of GPR120, GPR40, and vATPase E subunit in total cell extracts were determined by western blot analysis. Actin was used as a loading control. **b** Levels of vATPase E subunit in cytosolic and membrane fractions were determined by western blot analysis. Aldolase A and Na^+^/K^+^-ATPase were used as markers of cytosolic and membrane fractions, respectively

To investigate whether cerulein induces vATPase activation in AR42J cells, the levels of the vATPase V1 domain (E subunit) in the cytosol and membrane fractions were determined using an antibody against the vATPase E subunit. Cerulein did not change vATPase E subunit levels in total cell extracts (Fig. 1a). However, as shown in Fig. 1b, cerulein increased the levels of the vATPase E subunit in the membrane fraction but decreased its levels in the cytosolic fraction of AR42J cells. Levels of the cytosolic marker, aldolase A, and the membrane marker, Na^+^/K^+^-ATPase, were not changed by cerulein. These results indicate that cerulein induces the translocation of the V1 domain (E subunit) to the membrane in AR42J cells. In addition, GPR120, an omega-3 fatty acid receptor, and GPR40, a free fatty acid receptor 1, were not affected by cerulein treatment (Fig. 1a).

### DHA inhibited the cerulein-induced activation of vATPase in AR42J cells

**Fig. 2:**
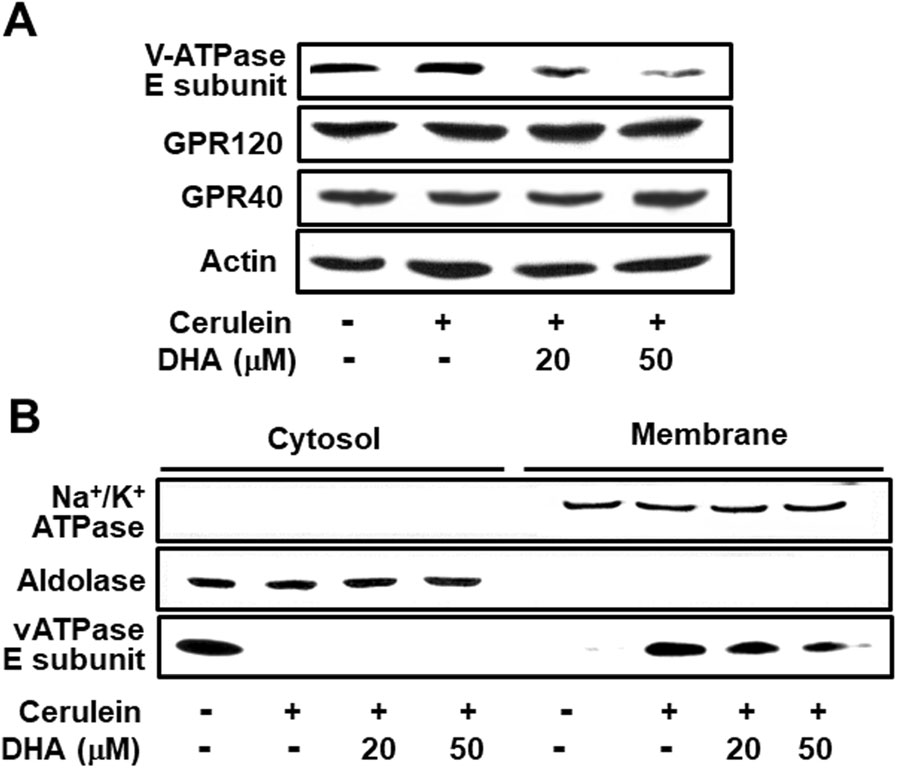
DHA decreased the activation of vATPase in cerulein-stimulated AR42J cells. Cells were pretreated with the indicated concentrations of DHA for 2 h and were then stimulated with cerulein (10^−8^ M) for 30 min. **a** Levels of GPR120 and GPR40 and the vATPase E subunit in total cell extracts were determined by western blot analysis. Actin was used as a loading control. **b** Levels of the vATPase E subunit in cytosolic and membrane fractions were determined by western blot analysis. Aldolase A and Na^+^/K^+^-ATPase were used as markers of cytosolic and membrane fractions, respectively

To determine the effect of DHA on cerulein-induced translocation of the vATPase E subunit to the membrane, the levels of the vATPase E subunit in cytosolic and membrane fractions were determined by western blot analysis. Pretreatment with DHA decreased the vATPase E subunit levels in total cell extracts (Fig. 2a). As shown in Fig. 2b, the cerulein-induced increase in vATPase E subunit levels in membrane fractions was suppressed by DHA. These results suggest that DHA decreases the expression of the vATPase E subunit and the activation of vATPase in cerulein-treated AR42J cells. GPR120 and GPR40 levels were not affected by cerulein treatment alone or following DHA pretreatment.

### DHA inhibited the cerulein-induced activation of zymogens in AR42J cells

**Fig. 3:**
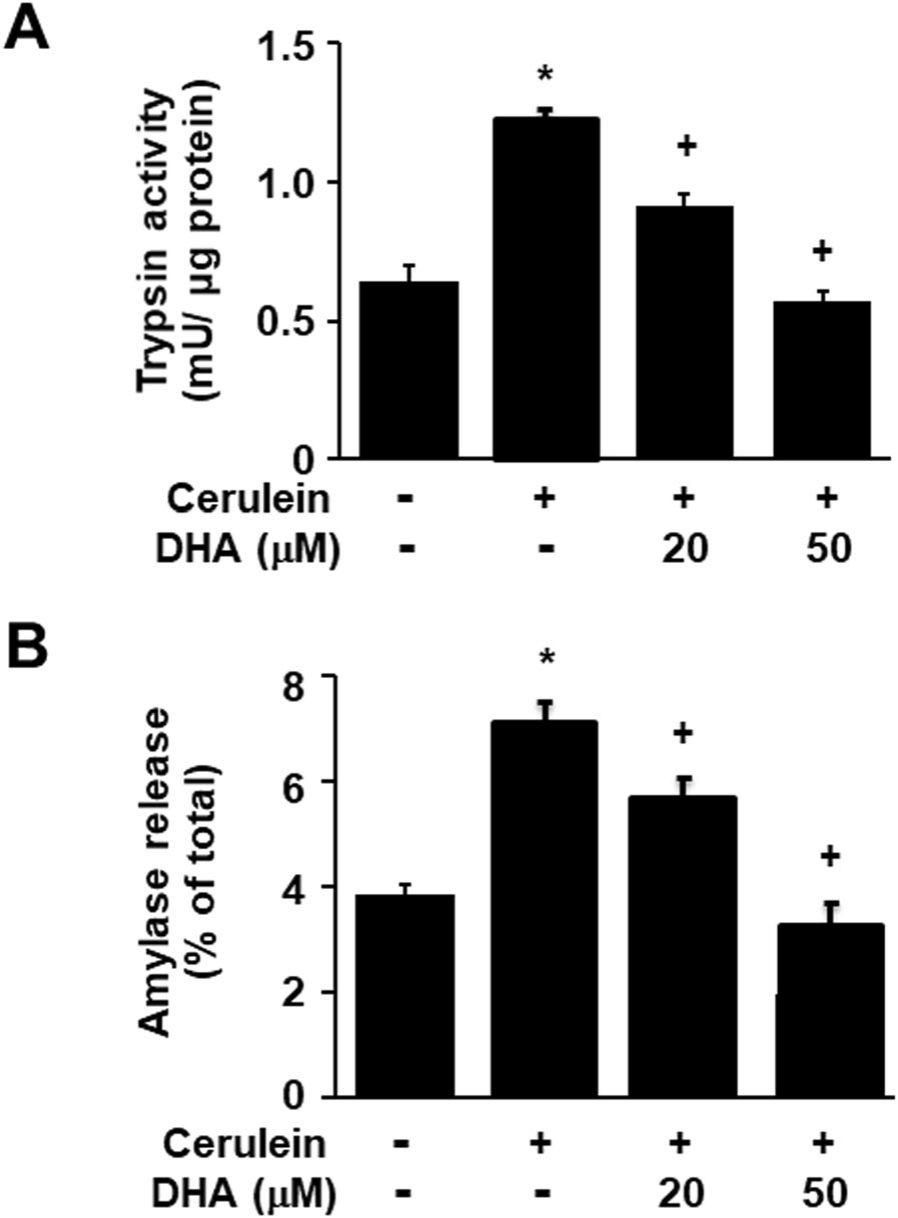
DHA suppressed cerulein-induced activation of zymogens in AR42J cells. Cells were pretreated with the indicated concentrations of DHA for 2 h and were then stimulated with cerulein (10^−8^ M) for 2 h. **a** Trypsin activity was assayed using a fluorogenic assay with a trypsin-specific substrate. Trypsin activity was expressed as milliunits per microgram protein. **b** The amount of secreted amylase was determined in the media, and total amylase activity was determined in cells using an Amylase Activity Colorimetric Assay kit. Amylase release was expressed as the percentage of secreted amylase/total amylase activity. Data were expressed as the mean ± S.E. of three independent experiments. *p < 0.05 vs. cells without any stimulation or treatment, ^+^*p* < 0.05 vs. cells with cerulein stimulation alone

As shown in Fig. 3, cerulein stimulation increased trypsin activity and amylase release, which are markers of zymogen activation in pancreatic acinar cells. DHA decreased trypsin activity and amylase release in cerulein-stimulated cells in a dose-dependent manner (Fig. 3a, b).

### GPR120 and GPR40 antagonist suppressed the inhibitory effect of DHA on zymogen activation in cerulein-stimulated AR42J cells

**Fig. 4:**
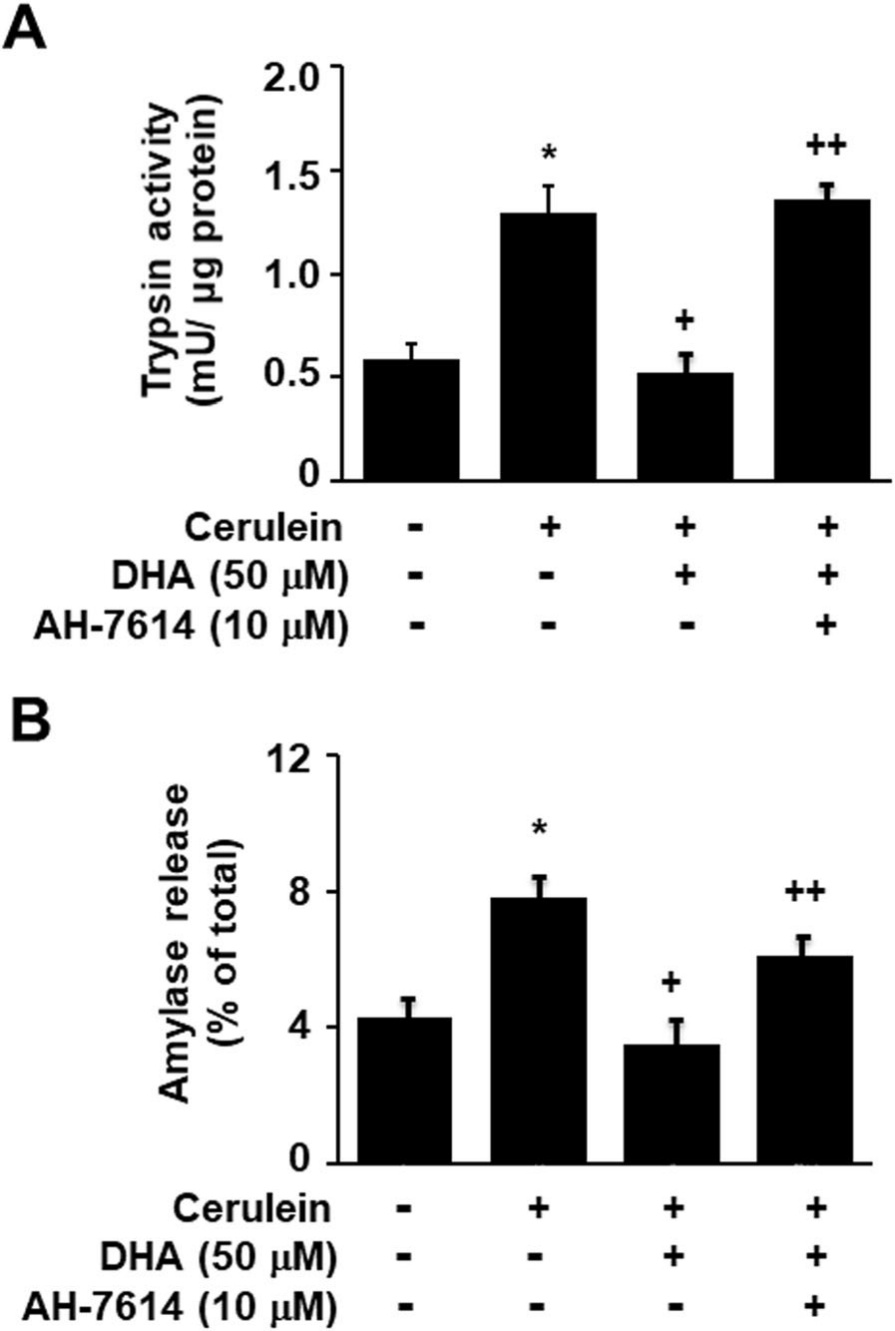
AH-7614 suppressed the inhibitory effect of DHA on zymogen activation in cerulein-stimulated AR42J cells. Cells were pretreated with 50 μM of DHA and 10 μM of AH-7614 for 2 h and were then stimulated with cerulein (10^−8^ M) for 2 h. **a** Trypsin activity was assayed using a fluorogenic assay with a trypsin-specific substrate. Trypsin activity was expressed as milliunits per microgram protein. **b** The amount of secreted amylase was determined in the media, and total amylase activity was determined in cells using an Amylase Activity Colorimetric Assay kit. Amylase release was expressed as the percentage of secreted amylase/total amylase activity. Data were expressed as the mean ± S.E. of three independent experiments. *p < 0.05 vs. cells without any stimulation or treatment, ^+^*p* < 0.05 vs. cells with cerulein stimulation alone, ^++^*p* < 0.05 vs. cells treated with cerulein and DHA

**Fig. 5:**
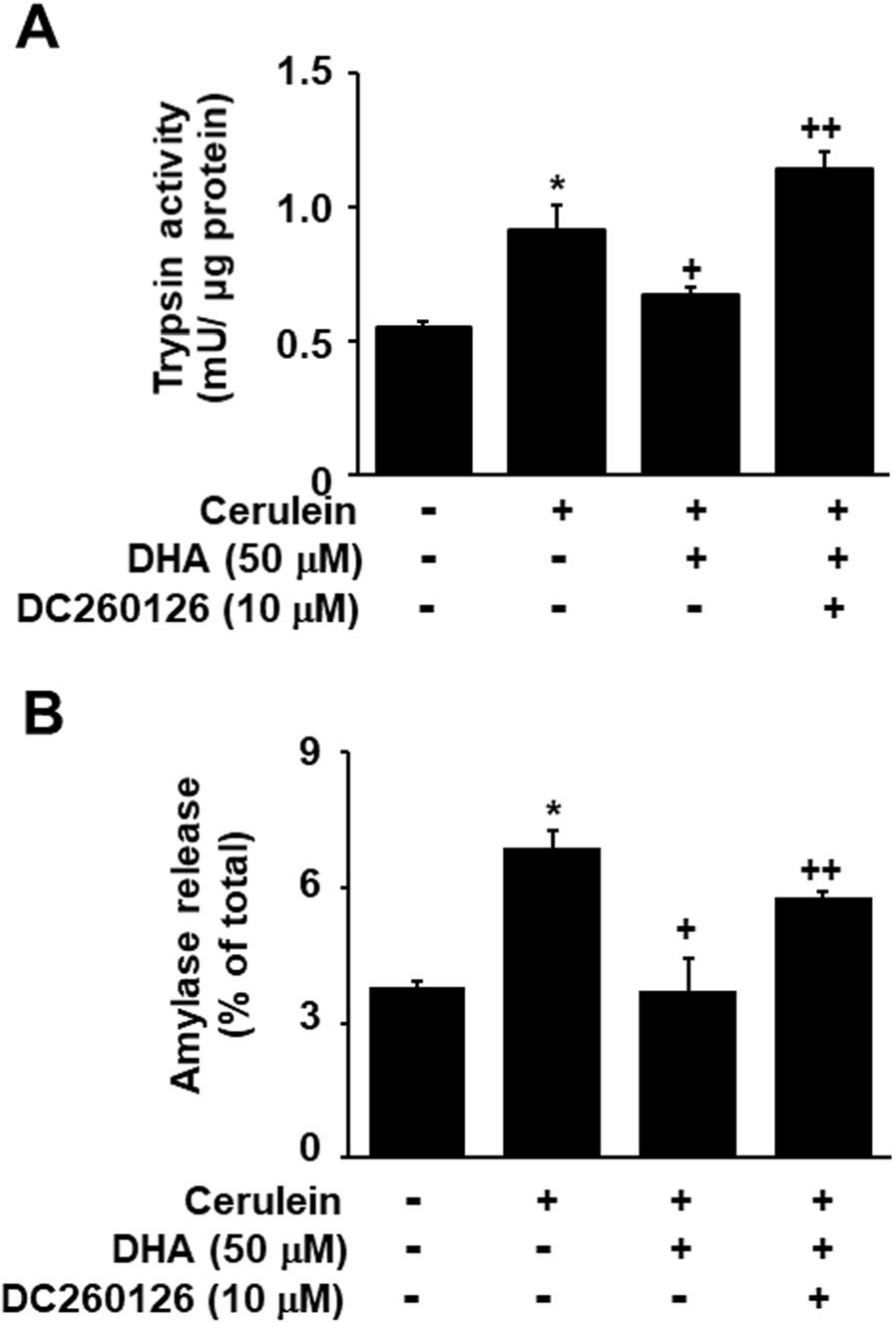
DC260126 reversed the inhibitory effect of DHA on cerulein -induced activation of zymogen in AR42J cells. Cells were pretreated with 50 μM of DHA and 10 μM of DC260126 for 2 h and were then stimulated with cerulein (10^−8^ M) for 2 h. **a** Trypsin activity was assayed using a fluorogenic assay with a trypsin-specific substrate. Trypsin activity was expressed as milliunits per microgram protein. **b** The amount of secreted amylase was determined in the media, and total amylase activity was determined in cells using an Amylase Activity Colorimetric Assay kit. Amylase release was expressed as the percentage of secreted amylase/total amylase activity. Data were expressed as the mean ± S.E. of three independent experiments. *p < 0.05 vs. cells without any stimulation or treatment, ^+^*p* < 0.05 vs. cells with cerulein stimulation alone, ^++^*p* < 0.05 vs. cells treated with cerulein and DHA

To elucidate the mechanism underlying the inhibitory effect of DHA on cerulein-induced zymogen activation via the GPR120 and GPR40 signaling pathway, we investigated whether a GPR120 antagonist AH-7614 and a GPR40 antagonist DC260126 could suppress this inhibitory effect of DHA. As shown in Fig. 4, cerulein stimulation increased zymogen activation, and this was inhibited by DHA. However, AH-7614 suppressed the inhibitory effect of DHA on cerulein-induced zymogen activation. In addition, the inhibitory effect of DHA on cerulein-induced zymogen activation was also suppressed by DC260126 (Fig. 5). These results demonstrate that DHA inhibits zymogen activation via GPR120 or GPR40-mediated pathway in cerulein-stimulated AR42J cells.

### DHA decreased vATPase in membrane fractions by ubiquitination and degradation of vATPase

**Fig. 6:**
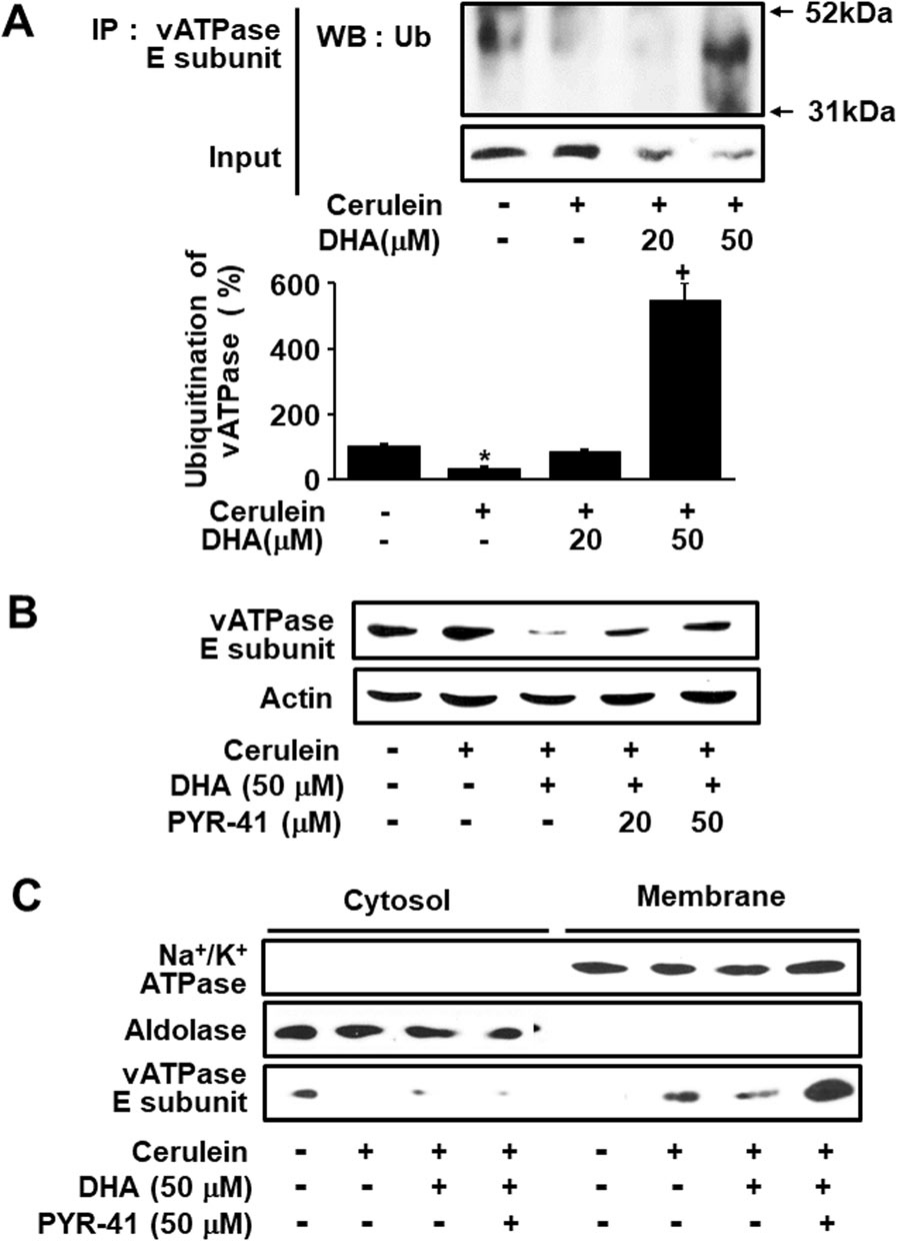
DHA decreased the levels of vATPase via ubiquitination in AR42J cells. **a** Cells were pretreated with the indicated concentrations of DHA for 2 h and were then stimulated with cerulein (10^−8^ M) for 30 min. Total cell extracts were prepared and subjected to immunoprecipitation (IP) analysis with anti-vATPase E subunit antibody, followed by western blotting (WB) with an anti-ubiquitin (Ub) antibody. Input was used as a control for protein expression determined by western blotting. The upper lane shows total cell extracts subjected to IP with an anti-vATPase E subunit antibody, followed by WB with an anti-ubiquitin antibody. The lower lane shows the amount of input vATPase E subunit determined with western blot analysis (upper panel). The level of the band densities corresponding to ubiquitinated vATPase E subunit to that of its input control was calculated. The band density level of “None” was set as 100%. Values are expressed as the mean ± S.E. of three/each group. *p < 0.05 vs. cells without any stimulation or treatment, ^+^*p* < 0.05 vs. cells with cerulein stimulation alone (lower panel). **b** Cells were pretreated with DHA (50 μM) and the ubiquitination inhibitor, PYR-41 (20 or 50 μM), for 2 h and were then stimulated with cerulein (10^−8^ M) for 2 h. Protein levels of the vATPase E subunit were determined by western blotting. Actin was used as a loading control. **c** Levels of the vATPase E subunit in cytosolic and membrane fractions were determined by western blot analysis. Aldolase A and Na^+^/K^+^-ATPase were used as markers of cytosolic and membrane fractions, respectively

To investigate whether DHA induced the ubiquitination of vATPase, cellular proteins were subjected to immunoprecipitation with an anti-ubiquitin antibody, followed by western blotting detecting of the E subunit of vATPase (Fig. 6a). DHA increased the interaction of the vATPase E subunit with ubiquitin. Amount of input vATPase E subunit was determined by western blotting at the same time as the control. To determine whether DHA induced the ubiquitination and degradation of vATPase, we investigated the effect of the ubiquitination inhibitor, PYR-41, on the membrane levels of the vATPase E subunit. As shown in Fig. 6b, PYR-41 suppressed the DHA-induced degradation of the vATPase E subunit. Furthermore, the DHA-induced decrease in membrane expression of the vATPase E subunit was suppressed by PYR-41 in cerulein-stimulated AR42J cells (Fig. 6c). These data indicate that DHA may induce the ubiquitination and degradation of membrane vATPase, resulting in the degradation of vATPase. The cytosolic marker, aldolase A, and the membrane marker, Na^+^/K^+^-ATPase, were not affected by any of these treatments.

### The ubiquitination inhibitor, PYR-41, suppressed the inhibitory effect of DHA on zymogen activation in cerulein-stimulated AR42J cells

**Fig. 7:**
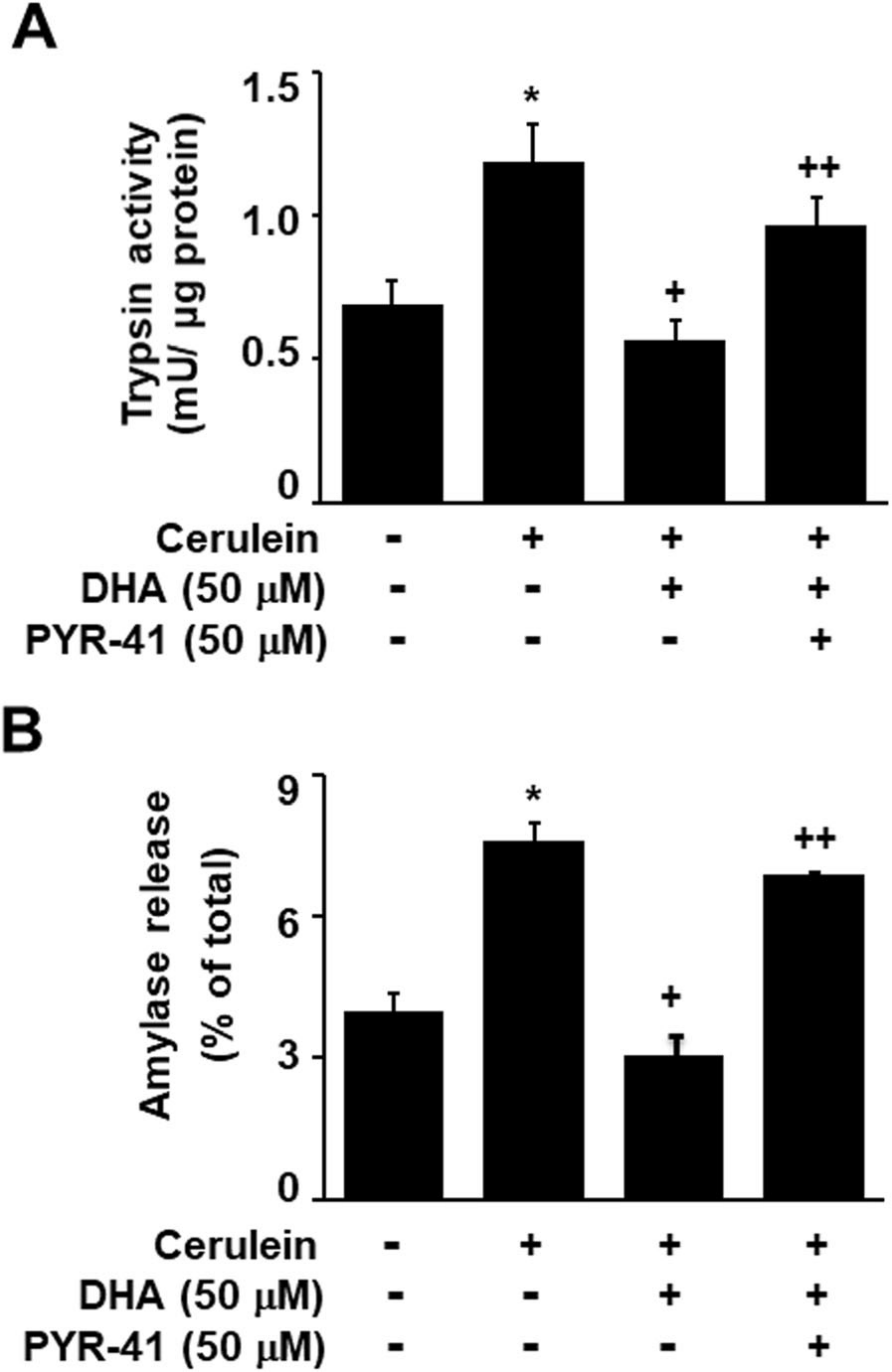
PYR-41 suppressed the inhibitory effect of DHA on zymogen activation in cerulein-stimulated AR42J cells. Cells were pretreated with DHA (50 μM ) and/or PYR-41 (50 μM) for 2 h and were then stimulated with cerulein (10^−8^ M) for 2 h. **a** Trypsin activity was assayed using a fluorogenic assay with a trypsin-specific substrate. Trypsin activity was expressed as milliunits per microgram protein. **b** The amount of secreted amylase was determined in the media, and total amylase activity was determined in cells using an Amylase Activity Colorimetric Assay kit. Amylase release was expressed as the percentage of secreted amylase/total amylase activity. Data were expressed as the mean ± S.E. of three independent experiments. *p < 0.05 vs. cells without any stimulation or treatment, ^+^*p* < 0.05 vs. cells with cerulein stimulation alone, ^++^*p* < 0.05 vs. cells treated with cerulein and DHA

To confirm the effect of DHA on the ubiquitination of vATPase, we investigated whether the ubiquitination inhibitor, PYR-41, could suppress the inhibitory effect of DHA on cerulein-induced zymogen activation in AR42J cells. As shown in Fig. 7, DHA inhibited cerulein-induced zymogen activation. However, PYR-41 suppressed the inhibitory effect of DHA on zymogen activation in cerulein-stimulated AR42J cells. These results suggest that DHA induces the ubiquitination and degradation of vATPase and suppresses zymogen activation in cerulein-stimulated AR42J cells.

### DHA increased the protein levels of Parkin in AR42J cells

**Fig. 8:**
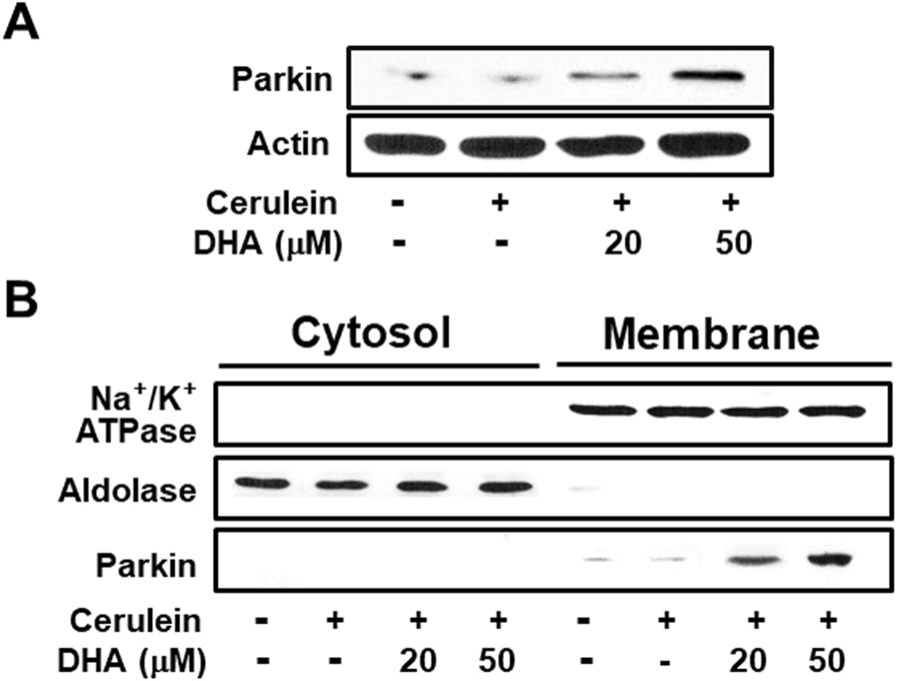
DHA increased the levels of Parkin in cerulein-stimulated AR42J cells. Cells were pretreated with the indicated concentrations of DHA for 2 h and were then stimulated with cerulein (10^−8^ M) for 30 min. **a** Levels of Parkin in total cell extracts were determined by western blot analysis. Actin was used as a loading control. **b** Levels of Parkin in cytosolic and membrane fractions were determined by western blot analysis. Aldolase A and Na^+^/K^+^-ATPase were used as markers of cytosolic and membrane fractions, respectively

Since vATPase is ubiquitinated by Parkin, which is a component of the multiprotein E3 ubiquitin ligase complex, we performed western blotting to investigate whether DHA increases the expression of Parkin. As shown in Fig. 8a, DHA increased the levels of Parkin in cerulein-stimulated AR42J cells. Furthermore, Parkin levels in membrane fractions were increased by DHA in cerulein-stimulated cells (Fig. 8b), indicating that DHA-induced Parkin is localized to the membrane. These results demonstrate that the ubiquitination and degradation of membrane vATPase may have been mediated by Parkin, which is upregulated by DHA in cerulein-stimulated cells.

## Discussion

The main role of the exocrine pancreas is to synthesize and secrete large amounts of digestive enzymes as inactive precursor zymogens. In acinar cells, these enzymes exist in inactive states within zymogen granules due to protease inhibitors, resulting in the inhibition of premature and intracellular activation of these digestive enzymes [27, 28]. However, when physiological conditions worsen, pancreatic autodigestion is initiated by premature zymogen activation, and acute pancreatitis can develop [29]. Therefore, premature zymogen activation is a key mediator in pancreatic inflammation.

DHA has been reported to show greater anti-oxidant and anti-inflammatory activities than other omega-3 PUFAs in renal epithelial cells and macrophages [30]. However, the molecular mechanisms underlying the anti-inflammatory effects of DHA in acute pancreatitis have not been well established. Lei et al. [31] assessed whether treatment with omega-3 PUFAs provide benefits to patients with acute pancreatitis based on literature database. They found that omega-3 PUFAs treatment resulted in a significantly reduced risk of mortality, infectious complications, and length of hospital stay. Previously, we reported that pretreatment with DHA reduced the cerulein-induced activation of nuclear factor kappa-light-chain-enhancer of activated B cells (NF-κB), protein kinase C δ, and interleukin (IL)-6 in pancreatic tissues of rats [32]. DHA suppressed pancreatic edema and decreased lipid peroxide levels, myeloperoxidase activity, and inflammatory cell infiltration into the pancreatic tissues of cerulein-treated rats.

We also reported that DHA suppressed the expression of inflammatory cytokines (IL-1β, IL-6) and inhibited the activation of transcription factor activator protein-1 in cerulein-stimulated pancreatic acinar cells [33]. These results suggest that DHA may be beneficial for preventing oxidative stress-induced pancreatic inflammation by inhibiting inflammatory cytokine expression in pancreatic acinar cells. Moreover, we recently showed that DHA induces peroxisome proliferator-activated receptor γ (PPARγ) activation and catalase expression, which inhibits ROS-mediated activation of Janus kinase 2 (JAK2)/signal transducer and activator of transcription 3 (STAT3) and IL-6 expression in cerulein-stimulated pancreatic acinar cells [34]. However, there has been no report regarding the effects of DHA on zymogen activation in the pathogenesis of acute pancreatitis. In the present study, we found that DHA decreased trypsin activity and amylase release in cerulein-stimulated AR42J cells. Moreover, the present results showed that DHA suppresses zymogen activation via GPR120- and GPR40-mediated pathway in AR42J cells.

Regarding zymogen activation in acute pancreatic inflammation, it has been reported that vATPase plays a central role in activating premature zymogens [35, 36]. Since vATPase acidifies the intracellular compartment, it is a candidate zymogen activator in pancreatic acini. Thus, we investigated whether DHA affects vATPase activity to inhibit zymogen activation in pancreatic acinar cells stimulated with cerulein. We found that cerulein induced the translocation of the vATPase V1 domain (E subunit) to membranes, which indicated vATPase activation. Furthermore, DHA decreased the levels of subunit E of the V1 domain in total cell extracts and in membrane fractions.

As previously mentioned, DHA induces the degradation of certain proteins by activating the ubiquitin-proteasome system [25, 26]. In the present study, DHA increased the interaction of vATPase with ubiquitin in cerulein-stimulated cells. The DHA-induced decrease in vATPase (subunit E) levels was suppressed by a specific ubiquitin E1 inhibitor, PYR-41, in cerulein-stimulated cells. In addition, PYR-41 suppressed the inhibitory effect of DHA on cerulein-induced zymogen activation in pancreatic acinar cells. These results strongly suggest that DHA inhibits zymogen activation by inducing the ubiquitination of vATPase in cerulein-stimulated AR42J cells.

An interesting finding in the present study was the mechanism employed by DHA to induce the ubiquitination of vATPase. In recent years, Parkin has attained much attention in Parkinson’s disease research. Several studies have demonstrated that Parkin, which is an E3 ubiquitin ligase, leads to the ubiquitination of multiple proteins [37, 38]. Thus, we investigated whether DHA induces Parkin to decrease vATPase in AR42J cells stimulated with cerulein. Indeed, DHA was found to increase Parkin levels in the membrane fractions of cerulein-stimulated AR42J cells, suggesting that DHA may have induced the ubiquitination of membrane vATPase by upregulating Parkin in cerulein-stimulated cells.

Ubiquitin ligases have been largely thought to be constitutively active. Parkin is also known to be constitutively active due to its autoubiquitination [39, 40]. Therefore, ubiquitination of vATPase may occur in untreated cells, leading to its degradation. Piplani et al. [41] demonstrated that cerulein treatment reduces Parkin expression and increases the translocation of Parkin to mitochondria in pancreas. They demonstrated that cerulein decreases the effect of Parkin on ubiquitination of vATPase in membrane. In the present study, as shown in upper panel of Fig. 6a, untreated cells showed ubiquitination of vATPase while cerulein-treated cells reduced the interaction of the vATPase E subunit with ubiquitin. However, treatment of DHA (at 50 μM) in the presence of cerulein significantly increased the interaction of the vATPase E subunit with ubiquitin.

As shown in lower panel of Fig. 6a, we compared the level of the band density corresponding to ubiquitinated vATPase E subunit to that of its input control. The band density level of “None” was set as 100%. Cerulein reduced ubiquitination of vATPase in AR42J cells. Treatment of DHA in the presence of cerulein increased ubiquitinated vATPase E subunit, which may be caused by upregulation of Parkin (as shown in Fig. 8) in AR42J cells.

Several studies reported that the cerulein significantly induces zymogen activation after 1 or 2 h in AR42J cells and isolated pancreatic acinar cells [42, 43]. Therefore, 2-h time point was used for determination of zymogen activation (trypsin activity, amylase release) in the present study. Further study should be performed whether cerulein treated with or without DHA affects expression of Parkin and activation of vATPase at 2-h culture.

In addition, Le Petit-Thévenin et al. [44] reported that treatment of DHA (50 and 100 μM) alone for 3 days did not significantly affect amylase release in AR42J cells. It is necessary to determine whether DHA alone changes trypsin activity and amylase release in AR42J cells for the future study.

A selective GPR120 antagonist AH-7614 and GPR antagonist DC 260126 suppressed the DHA-induced decrease in trypsin activity and amylase release in cerulein-stimulated cells. These results demonstrate that DHA inhibits zymogene activation, via GPR120 and GPR40, in pancreatic acinar cells stimulated with cerulein. Taken together, DHA upregulates Parkin which reduces vATPase-mediated zymogen activation, via GPR120 and GPR40, in cerulein-stimulated pancreatic acinar cells.

Ubiquitination is known to be constitutively active. Several studies report that PYR-41 alone treatment inhibited constitutive ubiquitination and degradation of proteins such as p53 and IκB in several cell lines [45, 46]. Therefore, treatment of PYR-41 alone may increase expression of membrane vATPase by inhibiting ubiquitination in cells. Moreover, there is no report about the effect of DC260126 on zymogen activation in pancreatic acinar cells. Therefore, further study should be performed to investigate whether treatment of PYR-41 alone or DC260126 alone affects activation of vATPase and zymogen in pancreatic acinar cells.

The limitation of the present study is that we only used one cell line in the present study; more cell lines and primary cells should be used to validate the present results in the future studies. In addition, to investigate the inhibitory effect and mechanism of DHA on cerulein-induced zymogen activation, further studies should be performed using in vivo experimental models.

## Conclusions

**Fig. 9:**
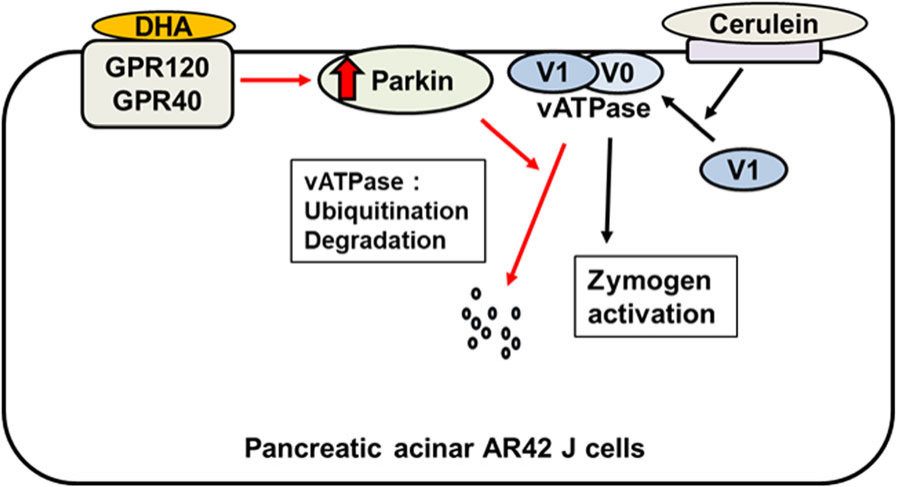
Schematic pathways for inhibitory effect of DHA on cerulein-induced activation of zymogen in AR42J cells. Cerulein induces the translocation of the cytosolic V1 domain of vATPase to the membrane, which may assemble with the V0 domain to activate vATPase in pancreatic acinar cells. vATPase activation may result in zymogen activation in pancreatic acinar cells. DHA binds to GPR120 and GPR40 which increases the level of Parkin in membrane. Parkin may induce the ubiquitination and degradation of membrane vATPase. Thus, DHA suppresses vATPase-mediated zymogen induced by cerulean. In summary, DHA inhibits zymogen activation, via GPR120 and GPR40, in pancreatic acinar AR42J cells stimulated with cerulein. Red arrows represent the effect of DHA. Black arrows mean the effect of cerulein stimulation

Cerulein induces the translocation of the vATPase V1 domain (E subunit) to membranes, which may assemble with the V0 domain to activate vATPase in pancreatic acinar cells. vATP activation induces zymogen activation in cerulein-stimulated pancreatic acinar cells. DHA may bind to GPR 120 and GPR 40 and increases the level of Parkin which may induce the ubiquitination and degradation of membrane vATPase in cerulein-stimulated pancreatic acinar cells. Thus, DHA suppresses vATPase-mediated zymogen activation that leads to decreasing trypsin activity and amylase release induced by cerulein in pancreatic acinar cells. As illustrated in Fig. 9, DHA inhibits zymogen activation by upregulating Parkin and suppressing vATPase, via GPR120 and GPR40, in cerulein-stimulated pancreatic acinar cells.

## Methods

### Reagents

DHA, a GPR120 antagonist AH-7614, an ubiquitination inhibitor PYR-41, and cerulein were purchased from Sigma-Aldrich (St. Louis, MO, USA). A GPR40 antagonist DC260126 was purchased from Cayman Chemical (Ann Arbor, MI, USA). DHA was dissolved in 0.5 M ethanol, while AH-7614, DC260126, and PYR-41 were dissolved in DMSO. Cerulein was dissolved in PBS containing 0.1% BSA, aliquoted, and stored at – 20 °C.

### Cell culture

Rat pancreatic acinar AR42J cells (pancreatoma, ATCC CRL 1492) were obtained from the American Type Culture Collection (Manassas, VA, USA) and cultured in Dulbecco’s modified Eagle’s medium (Sigma) supplemented with 10% fetal bovine serum (GIBCO-BRL, Grand Island, NY, USA) and antibiotics (100 U/mL penicillin and 100 μg/mL streptomycin). Cells were cultured at 37 °C in a humidified atmosphere of 95% air and 5% CO_2_.

### Experimental protocol

Cells (1 × 10^5^/mL) were pretreated with DHA (20 or 50 μM), with or without AH-7614 (10 μM), DC260126 (10 μM), or PYR-41(20 or 50 μM) for 2 h and then stimulated with cerulein (10^-8^ M) for 30 min (for the determination of vATPase, GPR120, and GPR40 levels, and immunoprecipitation to assess the vATPase and ubiquitin interaction) or 2 h (for the determination of trypsin activity and amylase release). The concentrations of AH-7614, DC260126, and PYR-41 and the culture periods used in the present study were adapted from previous studies [47–49].

### Preparation of cell extracts

Cells were harvested using trypsin-EDTA and pelleted by centrifugation at 1000×*g* for 5 min. Cell pellets were resuspended in lysis buffer containing 10 mM Tris, pH 7.4; 1% NonidetP-40 (NP-40); and a commercial protease inhibitor complex (Complete; Roche, Mannheim, Germany). Cells were lysed by drawing the cell suspension through a 1-mL syringe with several rapid strokes. The mixture was then incubated on ice for 30 min and centrifuged at 13,000×*g* for 15 min. Supernatants were collected and used as total cell extracts [50]. For subcellular fractionation, cell pellets were homogenized on ice with 300 μL of homogenization buffer containing 0.3 M sucrose, 10 mM HEPES (pH 7.4), and a commercial protease inhibitor complex. They were then subjected to low-speed centrifugation (500×*g*) for 10 min at 4 °C to remove nuclei and intact cells. The resulting post-nuclear supernatants were subjected to high-speed centrifugation (1,000,000×*g*) for 30 min at 4 °C in an Optima TLX Ultracentrifuge (Beckman Coulter Life Sciences, Indianapolis, IN, USA) to separate cytosolic and membrane fractions. The resulting supernatants were used as cytosolic extracts, and the pellets were resuspended in 70 μL of homogenization buffer and used as membrane fractions. Protein concentrations were determined using the Bradford assay (Bio-Rad Laboratories, Hercules, CA, USA).

### Western blot analysis

Whole-cell extracts (5~30 μg) were separated by 8–12% SDS-polyacrylamide gel electrophoresis, under reducing conditions and transferred onto nitrocellulose membranes (Amersham, Inc., Arlington Heights, IL, USA) by electroblotting. Protein transfer was verified using reversible staining with Ponceau S. Membranes were blocked with 3% non-fat dry milk in TBS-T (Tris-buffered saline and 0.2% Tween 20) for 1 h at room temperature. Proteins were detected using antibodies against vATPase E subunit (#PA5-29899; Thermo Fisher Scientific, Rockford, IL, USA), GPR120 (H-155; sc-99105; Santa Cruz Biotechnology, Dallas, TX, USA), GPR40 (SAB4501280, Sigma-Aldrich), aldolase A (sc-12059, Santa Cruz Biotechnology), Na^+^/K^+^-ATPase (sc-21712, Santa Cruz Biotechnology), and actin (sc-1615, Santa Cruz Biotechnology). Membranes were incubated overnight at 4 °C with primary antibodies diluted in TBS-T containing 3% non-fat dry milk. After washing with TBS-T, primary antibodies were detected with horseradish peroxidase-conjugated secondary antibodies (anti-rabbit, anti-mouse, or anti-goat) and visualized using an enhanced chemiluminescence detection system (Santa Cruz Biotechnology) after exposure to BioMax MR film (Kodak, Rochester, NY, USA). The target protein levels were compared to the levels of the loading control, actin.

### Determination of trypsin activity and amylase release

Trypsin activity was measured using a fluorogenic assay, with a substrate specific for trypsin (Boc-Glu-Ala-Arg-AMC; MQR-3135-v; Peptides International, Louisville, KY, USA). After the cells were treated with various agents, they were harvested using trypsin-EDTA and pelleted by centrifugation at 1000×*g* for 5 min. Cell pellets were washed with ice-cold PBS and then resuspended in lysis buffer containing 50 mM Tris, pH 8.0; 150 mM NaCl; 1 mM CaCl_2_; and 0.1% BSA. Cells were then lysed by homogenization and drawing the cell suspension through a 1-mL syringe with several rapid strokes. The mixture was then centrifuged at 13,000×*g* for 10 min. The substrate was added to the resulting supernatants to perform the assay. Trypsin activity was measured fluorometrically with excitation at 380 nm and emission at 440 nm. Fluorescence was calibrated using a trypsin standard curve.

The amount of secreted amylase was determined in the media, while total amylase activity was determined in cells using an Amylase Activity Colorimetric Assay kit (K711-100; Biovision, Milpitas, CA, USA). For the determination of total intracellular amylase, acinar cells were homogenized in lysis buffer containing 50 mM Tris, pH 8.0. Amylase release was expressed as the percentage of secreted amylase/total amylase activity [27, 51].

### Immunoprecipitation analysis of the vATPase and ubiquitin Interaction

Cells treated with or without DHA were lysed in 500 μM of immunoprecipitation buffer containing 10 mM Tris-HCl, pH 7.4; 100 mM NaCl; 1 mM EDTA; 1 mM EGTA; Complete protease inhibitors; 0.5% NP-40; 0.5% sodium deoxycholate; and 10% glycerol. Cells were then centrifuged at 15,000×*g* for 15 min. A polyclonal antibody and protein G-agarose were added to the cleared supernatant, and the resulting mixture was incubated overnight at 4 °C. The protein G-antibody-antigen complex was then collected by washing four times with immunoprecipitation buffer containing 150 mM NaCl; 10 mM Tris-HCl, pH 7.4; 1 mM EDTA; 1 mM EGTA; 0.5% NP-40; and 0.5% sodium deoxycholate. The final pellet was resuspended in 50 μL of SDS sample buffer and boiled for 10 min. This preparation was then subjected to western blot analysis. The level of the band densities corresponding to ubiquitinated vATPase E subunit to that of its input control was calculated. The band density level of “None” was set as 100%. Values are expressed as the mean ± S.E. of three/each group. A *p* value of 0.05 or less was considered statistically significant.

### Statistical analysis

All values were expressed as the mean ± S.E. of three independent experiments. Analysis of variance (ANOVA), followed by Newman-Keul’s post hoc test was used for statistical analysis. A *p* value ≤ 0.05 was considered statistically significant.

## Data Availability

All data generated or analyzed during this study are included in this published article.

## Abbreviations

CCK: Cholecystokinin
DHA: Docosahexaenoic acid
GPR: G-protein coupled receptor
PUFA: Polyunsaturated fatty acid
vATPase: Vacuolar ATPase
